# Morphological and Behavioral Effects in Zebrafish Embryos after Exposure to Smoke Dyes

**DOI:** 10.3390/toxics9010009

**Published:** 2021-01-10

**Authors:** Kimberly T. To, Lindsey St. Mary, Allyson H. Wooley, Mitchell S. Wilbanks, Anthony J. Bednar, Edward J. Perkins, Lisa Truong, Robyn L. Tanguay, Natàlia Garcia-Reyero

**Affiliations:** 1 Environmental Laboratory, US Army Engineer Research & Development Center, Vicksburg, MS 39180, USA; Kimberly.T.To@erdc.dren.mil (K.T.T.); Allyson.H.Wooley@erdc.dren.mil (A.H.W.); Mitchell.S.Wilbanks@erdc.dren.mil (M.S.W.); Anthony.J.Bednar@usace.army.mil (A.J.B.); Edward.J.Perkins@erdc.dren.mil (E.J.P.); 2 The Sinnhuber Aquatic Research Laboratory, Department of Environmental and Molecular Toxicology, The Sinnhuber Aquatic Research Laboratory, Oregon State University, Corvallis, OR 97333, USA; stmarylindsey@gmail.com (L.S.M.); Lisa.Truong@oregonstate.edu (L.T.); robyn.tanguay@oregonstate.edu (R.L.T.)

**Keywords:** anthraquinone dyes, Disperse Blue 14, Solvent Violet 47, zebrafish, behavior

## Abstract

Solvent Violet 47 (SV47) and Disperse Blue 14 (DB14) are two anthraquinone dyes that were previously used in different formulations for the production of violet-colored smoke. Both dyes have shown potential for toxicity; however, there is no comprehensive understanding of their effects. Zebrafish embryos were exposed to SV47 or DB14 from 6 to 120 h post fertilization (hpf) to assess the dyes’ potential adverse effects on developing embryos. The potential ability of both dyes to cross the blood–brain barrier was also assessed. At concentrations between 0.55 and 5.23 mg/L, SV47 showed a dose-dependent increase in mortality, jaw malformation, axis curvature, and edemas. At concentrations between 0.15 and 7.54 mg/L, DB14 did not have this same dose-dependence but had similar morphological outcomes at the highest doses. Nevertheless, while SV47 showed significant mortality from 4.20 mg/L, there was no significant mortality on embryos exposed to DB14. Regardless, decreased locomotor movement was observed at all concentrations of DB14, suggesting an adverse neurodevelopmental effect. Overall, our results showed that at similar concentrations, SV47 and DB14 caused different types of phenotypic effects in zebrafish embryos.

## 1. Introduction

Smoke dyes are synthetic dyes used to color smoke in pyrotechnic devices for a broad range of applications, including entertainment (i.e., special effects, fireworks), safety (i.e., distress signals and location markers), or military (i.e., signals and training). Many of those synthetic dyes are also used in other applications such as paper, plastics, leather, cosmetics, food, and textiles [1]. The industrial use of synthetic dyes has led to high levels of wastewater contamination that threaten aquatic environments [2,3]. Dyes have been detected in industrial effluents at varying concentrations, with general reports of 10–50 mg/L of dyes detected in effluents and reports of up to 300 mg/L from textile effluents [4,5]. It is difficult to quantify the amount of global dye consumption, but it is estimated that 700,000 tons of synthetic dyes are produced per year [6]. Regardless, the presence of dyes in wastewater is a pervasive issue that has led to safety concerns surrounding their discharge into the environment [6].

Among the most commonly used classes of dyes are azo and anthraquinone dyes, which make up approximately 70% and 15% of industrial dye consumption, respectively [7,8]. Compared to azo dyes, anthraquinone dyes are more difficult to degrade during wastewater treatment due to the stability of their chemical structure. Yet, toxicity information for azo dyes is more abundant than for anthraquinone dyes [9].

Anthraquinone dyes have been observed to induce toxicity at various trophic levels. For example, Reactive Blue 4 was shown to inhibit root growth and germination in common wheat (*Triticum aestivum*) and induce cytotoxicity and genotoxicity in human keratinocyte and fish epithelial cell lines at concentrations between 10 and 200 mg/L [10]. Disperse Blue 3 inhibited bacterial luminescence in *Vibrio fischeri* (EC50 = 488 mg/L), inhibited algal growth in *Selenastrum capricornutum* (EC50 = 0.5 mg/L), and significantly decreased the ingestion rate of *Tetrahymena pyriformis* at 500 mg/L [11]. Vat Green 3 induced yolk sac edema and swim bladder deflation in zebrafish at 100 mg/L [12]. Since anthraquinones are such a broad class of dyes, toxicity associated with these dyes has not been studied in detail [13] warranting the need for further testing.

Solvent Violet 47 (SV47) and Disperse Blue 14 (DB14) are two anthraquinone dyes that have been historically used in different dye mix formulations to produce violet-colored smoke. SV47 is now primarily used as an intermediate dye for the production of disperse and vat dyes [14]. DB14 is now used to color textiles and lubricants and has been identified in sewage treatment plant wastewater [2,15,16].

There has been increasing concern about the potential neurotoxic effects of synthetic dyes. For instance, some food coloring dyes have been linked to neurobehavioral alterations such as sleep disorders, hyperactivity, and even autism [17,18]. The developing nervous system and the blood-brain barrier (BBB) are particularly vulnerable to chemicals, with the early onset of exposure linked to many neurological disorders, including autism spectrum disorder, Alzheimer’s disease, and Parkinson’s disease [19].

The BBB is a physical barrier formed by astrocytes along with the cerebral microvascular endothelium, pericytes, neurons, and the extracellular matrix. It prevents many substances from entering the brain, through both physical (tight junctions) and metabolic (enzymes, diverse transport systems) barriers [20], thus protecting the brain against chemical substances [21]. Chemicals that can cross the BBB could potentially compromise the central nervous system and lead to neurotoxicity. For instance, lead, a well-known neurotoxicant, crosses the BBB and concentrates in the brain due to its ability to substitute for calcium ions [20]. Here, we tested the ability of SV47 and DB14 to cross the BBB in order to better understand their potential to induce neurotoxicity and neurodevelopment.

The structure of the early-developing zebrafish is similar to other vertebrates, with the advantage of being transparent, allowing for automated visual assessment of developmental outcomes [22,23]. Additionally, the development of the central nervous system and the BBB are also well conserved between zebrafish and other vertebrates [24,25,26]. Specifically, the formation of a functional BBB in zebrafish embryos has been observed at about 72 h post fertilization (hpf) [27,28], with maturation occurring until at least 10 days post fertilization (dpf) [29]. Due to these similarities, the rapid development of the brain, and its small size, the zebrafish is being increasingly used as a complementary model for in vivo neurotoxicity screening [19] and developmental toxicity testing. Here, we utilize the zebrafish embryo developmental toxicity assay to screen SV7 and DB14 for toxicity and report a comprehensive set of phenotypic outcomes.

## 2. Materials and Methods

Fertilized embryos (Tropical 5D) were selected and staged, according to Kimmel et al. [30]. The chorions of 4 hpf embryos were removed using 83 µL of 25.3 U/µL pronase (Roche, Indianapolis, In) using a custom automated dechorionator, as described by Mandrell et al. [31]. At 6 hpf, the embryos were transferred into individual wells of a round-bottom 96-well plate filled with 100 µL of embryo medium. The smoke dyes DB14 (Def Std 68-58/2) and SV47 (mil-D-3688) were provided by Walrus Enterprises LLC (Northampton, MA, USA) and dispensed into each well using an HP D300 Digital dispenser. Each dye was suspended in 100% DMSO and added to the single-use cassette wells of the D300 at 20 mM. After the dyes were dispensed, the wells were normalized with 0.64% DMSO. SV47 was tested at 0, 0.55, 1.20, 3.16, 4.20, and 5.23 mg/L, while DB14 was tested at 0, 0.15, 0.75, 1.69, 5.37, and 7.54 mg/L. For each of the dyes, embryos were exposed to 6 concentrations with 32 animals per concentration on two replicate plates. Afterwards, parafilm was placed between the lid and the wells to reduce evaporation. The plates were placed on an orbital shaker at 235 rpm at 28 °C for 16 h to create a homogenous test solution before being placed in a static incubator until 120 hpf.

After the chemical exposures were initiated, the embryos were kept in the dark until 24 hpf to test for embryonic photomotor response (EPR) [32]. The EPR assay is a behavioral assay that detects embryonic zebrafish spontaneous movement in response to visible light. Briefly, the assay consists of 3 phases: 30 s of darkness (Background), a pulse of intense visible light (13,000 lux), 9 s darkness (Excitation), a second pulse of visible light, and 10 s darkness (Refractory). Following the EPR assay, the embryos were evaluated for mortality and delays in progression. The plates were placed back into the incubator until 120 hpf. At 120 hpf, the embryos were subjected to a larval photomotor response assay (LPR) using the Viewpoint Zebrabox system (Viewpoint Life Sciences, Lyon, France). The assay was a total of 24 min, with a datapoint recorded every 6 s. The total distance was tracked for each of the 4 light-dark cycles, with the first cycle treated as an acclimation period and discarded from the analysis. Each cycle consisted of 3 min of alternating visible light (1000 lux) and dark (IR). Animals exhibiting morbidity or mortality were excluded from the analysis. After LPR, the embryos were assessed for a suite of malformations [18], which included yolk sac or pericardial edema, body axis, trunk length, caudal and pectoral fin, pigmentation, somite deformities, eye, snout, jaw, otolith malformations, gross brain development, notochord and circulating deformities, swim bladder presence and inflation, and the presence of a touch response. These effects were collected in a binary manner and stored in a laboratory information management system [22].

### 2.1. Analytical Chemistry

Samples arrived in the laboratory dissolved in DMSO and at varying volumes. Samples were further diluted in methylene chloride to reduce the effect of the DMSO solvent and analyzed using an Agilent 6890 Gas Chromatograph and 5973 Mass Spectrometer (GC-MS) equipped with a polymer (5% diphenyl/95%dimethylsiloxane) column measuring 30 m × 0.25 mm × 0.25 μm. The oven parameters were as follows: initial temperature of 40 °C held for 0.5 min, 10 °C/min to 100 °C, 25 °C/min to 280 °C held for 3 min, 5 °C/min to 300 °C held for 3 min, 25 °C/min to 325 °C. Instrumental control parameters, including mass spectrometer tuning, were performed following USEPA SW 846-Method 8270 guidelines. Calibration was achieved using a 5-point response curve and internal standards to adjust for instrument variability. Calibration standards were prepared using solid dyes provided by the supplier noted previously. Data reductions were performed using the Chemstation software (Agilent Technologies, Santa Clara, CA, USA).

### 2.2. Blood-Brain Barrier Permeability

The BBB-PAMPA Permeability Assay was performed by Creative Bioarray (Shirley, NY, USA) using a protocol based on Rabal et al. [33], with propranolol as the positive control. Both compounds were tested at a concentration of 10 μM. Briefly, the donor solutions of SV47 or DB14 (10 μM, 150 μL in PBS/DMSO 19:1) were added to each well of the donor plate, whose PVDF membrane was precoated with 5 μL of 1% brain polar extract (porcine)/dodecane mixture. Then, 300 μL of PBS was added to each well of the PTEF acceptor plate. The donor and acceptor plates were combined together and incubated for 4 h at room temperature with shaking at 300 rpm. In each plate, SV47 or DB14 and the positive control were tested in duplicate. After incubation, acceptor samples were prepared by mixing 270 μL of the solution from each acceptor well with 130 μL of acetonitrile containing the internal standard. Donor samples were prepared by mixing 20 μL of the solution from each donor well with 250 μL of PBS and 130 μL of acetonitrile containing the internal standard. Then, SV47 or DB14 concentrations in the acceptor and the donor wells were analyzed by LC-MS/MS. The permeability rate (*P_e_* in nm/s) was calculated with the following equation:

(1) $$P_{e}=C\;\times \;\left(-\ln\left(1-\frac{{\left[drug\right]}_{\mathrm{acceptor}}}{{\left[drug\right]}_{\mathrm{equilibrium}}}\right)\;\right)\times \;10^{7}$$

where $C=\left(\frac{VD\;\times \;VA}{\left(VD+VA\right)\times \;Area\;\times \;time)}\right)$;

[*drug*]_equilibrium_ = ([*drug*]_donor_ × *VD* + [*drug*]_acceptor_ × *VA*)/(*VD* + *VA*);
[*drug*]_acceptor_ = (Aa/Ai × DF)_acceptor_;
[*drug*]_donor_ = (Aa/Ai × DF)_donor_;
*VD* = 0.15 mL; *VA* = 0.30 mL; *Area* = 0.28 cm^2^; *time* = 14,400 s.
Aa/Ai: Peak area ratio of NAC and the internal standard; DF: Dilution factor (13.5).

Finally, the permeability of the tested compounds was classified by their *P_e_* as high (*P_e_* > 10 nm/s), moderate (1 < *P_e_* < 10), or low (*P_e_* < 1).

### 2.3. Statistical Analysis

Statistical analyses were conducted in R v.3.6.1 [34]. Morphological endpoints were compared between the treatment groups and the control group using Fisher’s Exact Test. The morphological Lowest Effect Limits (LELs) were identified as the lowest concentration eliciting a significant difference from control. Significance was defined by the Bonferroni-adjusted *p*-value (0.05/5 = 0.01), which was adjusted for the number of concentrations within each dye. The concentration at which 50% mortality (LC50) was observed and calculated using the drm function within the R drc package.

Behavior was analyzed separately for the light phase and dark phase. Within each phase, the distribution of average movement per fish was compared between the treatment and control using a Kolmogorov–Smirnov test. Behavioral LELs were identified as the lowest concentration eliciting a significant difference from the control. Significance was defined by the Bonferroni-adjusted *p*-value (0.05/5), which was adjusted for the number of concentrations within each dye.

## 3. Results and Discussion

### 3.1. Morphological Effects

**Figure 1:**
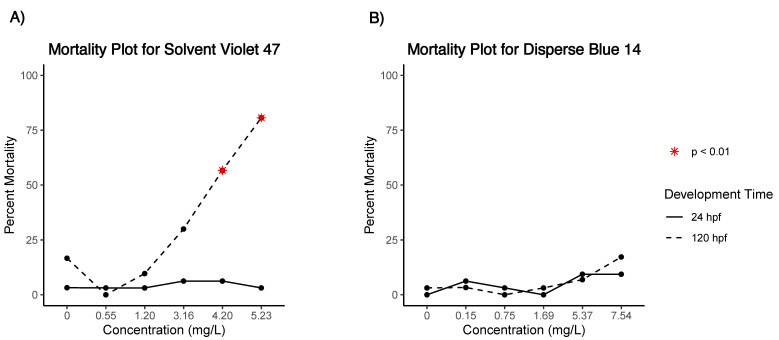
Summary of mortality at 24 h post fertilization (hpf) and 120 hpf in (**A**) Solvent Violet 47 and (**B**) Disperse Blue 14 treatment groups. * Indicates statistical significance (*p* < 0.01) against the control (0 mg/L).

**Figure 2:**
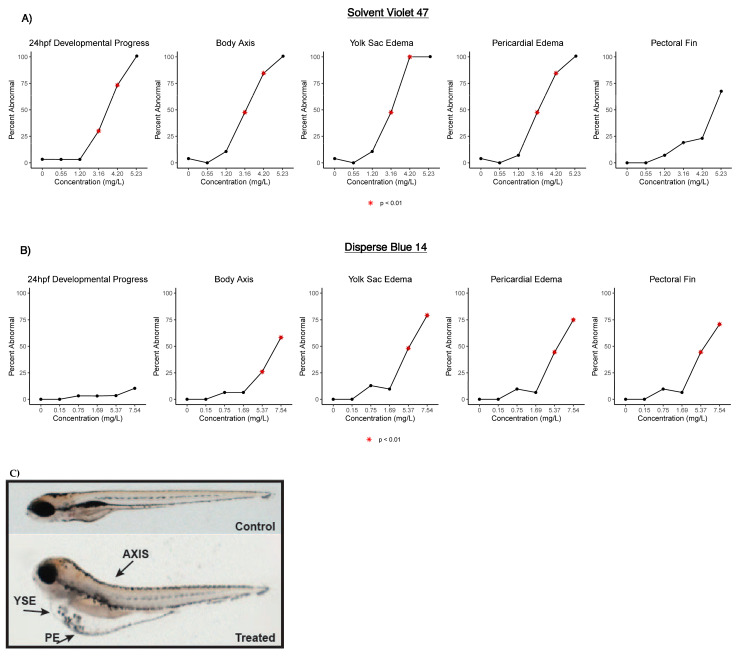
Summary of affected and unaffected selected morphological endpoints in (**A**) Solvent Violet 47 and (**B**) Disperse Blue 14 treatment groups. The figure shows the percentage of embryos per concentration of dye. * Indicates statistical significance (*p* < 0.01) against the control (0 mg/L); (**C**) representative endpoints assessed in embryos exposed to the smoke dyes.

One embryo from the SV47 control group was removed from analysis due to low well quality. Embryos exposed to SV47 showed a concentration-dependent increase in mortality with an LC_50_ of 4.37 mg/L (Figure 1a). Embryos exposed to 0.55 and 1.20 mg/L of SV47 had a morphology and behavior consistent with the control (Table 1 and Table 2). Developmental progression, axis curvature, jaw malformation, yolk sac edema, pericardial edema, and touch response had an LEL of 3.16 mg/L, with the 3.16 and 4.20 mg/L groups significantly different from the control. In addition, malformed snout and caudal fin have LELs of 4.20 mg/L (Table 1). The 5.23 mg/L treatment group only had six surviving embryos. Given the small postmortality sample size, we refer to the endpoints observed in all six of the surviving embryos: developmental progression, axis curvature, yolk sac edema, and pericardial edema (Figure 2a).

DB14 treatment groups showed no significant difference or dose–response in mortality between treatment groups and the control group (Table 1, Figure 1b). There was no significant effect in morphology in the 0.15, 0.75, or 1.69 mg/L treatment groups. Significantly different morphological endpoints were the same for the 5.37 and 7.54 mg/L treatment groups, with an LEL of 5.37 mg/L for yolk sac edema, axial curvature, eye malformation, snout malformation, jaw malformation, pericardial edema, and pectoral fin malformation (Table 1).

### 3.2. Behavioral Changes

**Figure 3:**
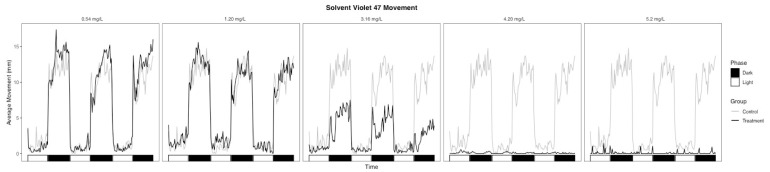
Average movement of Solvent Violet 47 treatment groups throughout the photomotor assay.

The light-phase movement of the SV47 3.16 mg/L treatment group was similar to the controls (Figure 3), but embryos had a subdued response to the phase change and significantly lower movement during the dark phase relative to the controls (Table 2, *p* = 0.002). Embryos in the 4.20 and 5.23 mg/L treatment groups showed almost no movement throughout the assay (Figure 3). The lack of movement in the dark phase observed in the higher doses may be due to physical defects rather than an indication of neurotoxicity given effects seen at lower concentrations [35]. One common phenotype that affects fish buoyancy and motility is impaired swim bladder inflation [36,37], with some textile dyes showing effects on swim bladder function [12,38]. However, no SV47 treatment groups had significant differences in swim bladder function (Table 1). Similarly, Abe et al. showed that zebrafish embryos exposed to large concentrations of the textile dye erythrostominone had no significant decrease in swim bladder function but had decreased dark-phase movement attributed to yolk sac edema and pericardial edema [39]. Our data show embryos in the 3.16, 4.20, and 5.23 mg/L SV47 treatment groups with high instances of yolk sac edema and pericardial edema, which could therefore be a physical obstruction inhibiting swim ability.

**Figure 4:**
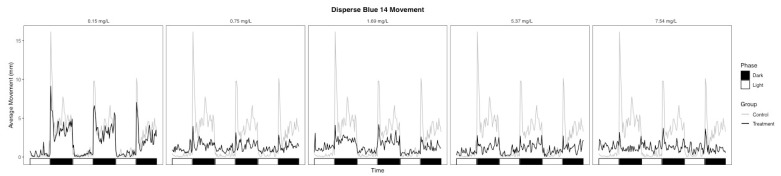
Average movement of Disperse Blue 14 treatment groups throughout the photomotor assay.

Although there were no significant changes in morphology in the 0.15, 0.75, and 1.69 mg/L DB14 treatment groups, the LEL for dark-phase movement was 0.15 mg/L, and the dark-phase movement was lower than the control across all treatment groups (Table 2, Figure 4). In particular, the 0.75 and 1.69 mg/L treatment groups showed a slight response to the phase change but lower movement throughout the dark phases (Figure 4). Touch response, an indicator of skeletal muscle function or the innervation of the nerves, was not significantly impaired for any DB14 treatment group (Table 1).

Contrary to the SV47 results, the 5.37 and 7.54 mg/L DB14 treatment groups were hyperactive during the light phase despite having high rates of yolk sac edema, bent axis, and pericardial edema (Table 2, Figure 2b). Because light-phase movement in the controls was low, it is possible that hyperactivity was observable for periods where lower movement is expected but periods where greater movement is expected to show the inhibitory effects of the malformations.

### 3.3. Analytical Chemistry

Analytical chemistry analysis was performed on the stocks used for the exposures. The results are summarized in Table 3.

### 3.4. Blood-Brain Barrier Permeability

The in vitro BBB model was moderately permeable to SV47 (1.586 nm/s, Table 3) and highly permeable to DB14 (26.259 nm/s, Table 4).

Interestingly, the highly permeable dye was the one that presented less morphological effects but more behavioral alterations. While hypoactivity can have many different causes (i.e., morphological deformities, secondary paralysis, impaired neurodevelopment, etc.), it is worth noting that morphological abnormalities were not detected with the DB14 treatment. These results suggest that assays to measure BBB permeability might be a useful complement to zebrafish embryo exposures, particularly when assessing neurodevelopmental endpoints. Understanding the capability of chemicals to cross the BBB can help discern potential neurotoxic effects on developing organisms.

## 4. Conclusions

Similar to other anthraquinone dyes, toxicity information for SV47 and DB14 is sparse. With the continued use of anthraquinone dyes in industrial and commercial settings, it is important to fill toxicological data gaps so that we can understand the potential adverse outcomes from their presence in wastewater and aquatic ecosystems. Here, we show that SV47 and DB14 are both found to cause toxic effects in zebrafish embryos. SV47 induced high mortality, with an LC_50_ of 4.37 mg/L, and high rates of morphological abnormalities. DB14 induced significant morphological effects at the highest tested concentrations and showed potential for neurotoxicity at all concentrations. Future assessments of SV47 and DB14 should use an integrative approach that combines these phenotypic markers and omics analyses to understand the molecular mechanisms of their toxicity. Our results showed that the zebrafish embryo is a useful model to understand the potential adverse effects of smoke dyes on vertebrates, particularly when paired with other complementary in vitro assays.

## Figures and Tables

**Table 1:** Lowest Effect Levels (LELs) and Fisher’s Exact Test (P) *p*-values for morphological endpoints. (-) indicate no significance at any concentration. At 24 hpf, embryos were assessed for mortality (MO24), developmental progression (DP24), spontaneous movement (S24), and notochord distortion (NC24). Additionally, at 120 hpf, embryos were assessed for mortality (MORT), and morphological malformations, including yolk sac edema (YSE), bent body axis (AXIS), eye (EYE), snout (SNOU), jaw (JAW), otic (OTIC), pericardial edema (PE), brain (BRAI), somite (SOMI), pectoral fin (PFIN), caudal fin (CFIN), circulation (CIRC), pigmentation (PIG), trunk length (TRUN), swim bladder (SWIM), notochord distortion (NC), and alterations in touch response (TR).

Endpoint	Solvent Violet 47		Disperse Blue 14	
	LEL	*p*	LEL	*p*
MO24	-	-	-	-
DP24	38.5	3.84 × 10^−5^	-	-
SM24	-	-	-	-
NC24	-	-	-	-
MORT	38.5	0.002	-	-
YSE	29	0.001	35.6	6.3 × 10^−6^
AXIS	29	0.001	35.6	0.003
EYE	48	0.0005	35.6	0.003
SNOU	38.5	0.003	35.6	0.007
JAW	29	0.006	35.6	0.007
OTIC	-	-	-	-
PE	29	0.001	35.6	1.95 × 10^−5^
BRAI	-	-	-	-
SOMI	-	-	-	-
PFIN	48	0.0005	35.6	1.95 × 10^−5^
CFIN	38.5	0.01	-	-
PIG	-	-	-	-
CIRC	-	-	-	-
TRUN	-	-	-	-
SWIM	-	-	-	-
NC	-	-	-	-
TR	29	0.006	-	-

**Table 2:** Lowest Effect Levels (LEL) and Kolmogorov–Smirnov (P) *p*-values for movement. Delta = (Average Treatment Movement − Average Control Movement)/(Average Control Movement).

Phase	Solvent Violet 47				Disperse Blue 14			
	LEL μM	*p*	Delta	Effect	LEL μM	*p*	Delta	Effect
Light	38.5	0.002	−0.915	Hypoactive	35.6	0.003	0.963	Hyperactive
Dark	29	0.002	−0.630	Hypoactive	1	0.007	−0.270	Hypoactive

**Table 3:** Analytical chemistry results showing nominal versus measured concentrations.

Dye	MW	Nominal (mM)	Measured (mM)	Measured (mg/L)
SV47	240.26	40	18.147	4360
DB14	266.29	40	22.64	6030

**Table 4:** Blood–brain barrier permeability assay results. Moderate permeability: 1 < *P_e_* < 10 nm/s. High permeability: *P_e_* > 10 nm/s.

Compound ID	Test Concentration	Incubation Time	Mean *P_e_* (nm/s)	% Mean Recovery	Permeability
Solvent Violet 47	10 μM	4 h	1.586	113.5	Moderate
Disperse Blue 14	10 μM	4 h	26.259	11.5	High

## Data Availability

The data presented in this study are available on request from the corresponding author.
